# RNA transcripts in salivary extracellular vesicle cargo isolated from aged populations

**DOI:** 10.3389/fragi.2025.1707720

**Published:** 2026-01-12

**Authors:** Sicheng Wen, Chang Yu, Maxfield M. G. Kelsey, Mandy Pereira, Hannah Alaimo, Em Teixeira, Jenna Pracht, Lori A. Daiello, Jonathan Drake, John M. Sedivy, Zhijin Wu, Peter Quesenberry, Jill A. Kreiling

**Affiliations:** 1 Department of Medicine, Division of Hematology/Oncology, Rhode Island Hospital, Brown University, Providence, RI, United States; 2 Department of Biostatistics, Brown University School of Public Health, Providence, RI, United States; 3 Department of Molecular Biology, Cell Biology, and Biochemistry, Brown Center on the Biology of Aging, Brown University, Providence, RI, United States; 4 Alzheimer’s Disease and Memory Disorders Center, Rhode Island Hospital, Providence, RI, United States; 5 Department of Neurology, Warren Alpert Medical School of Brown University, Providence, RI, United States

**Keywords:** extracellular vesicle, RNA, biomarker, alzheimer’s disease, neurodegeneration

## Abstract

**Introduction:**

Human saliva contains numerous factors, including DNA, RNA, and protein, that may reflect the health status of the individual. Many of these factors are contained within extracellular vesicles (EVs). The contents of EVs are thought to mirror the cytoplasm of the cell of origin, providing insight into the health of the cell. We investigated the RNA content from EVs isolated from saliva (salEVs) to determine if we could detect transcripts associated with neurodegenerative conditions.

**Methods:**

We characterized the RNA cargo of salEVs isolated from individuals over the age of 65 with normal cognition. The salEV RNA content was analyzed by RNA-seq and NanoString miRNA analysis.

**Results:**

We found approximately 48.4% of the reads mapped to the human genome, with the remainder mapping to prokaryotic genomes. The transcripts included protein-coding RNA, long non-coding RNA, retrotransposons, and miRNAs. A significant number of the protein-coding transcripts were associated with pathways involved in neurodegenerative conditions. In addition, there was an enrichment of transcripts containing AP-2ε, HEYL, HES4, and TCFL5 transcription factor binding sites. We found that the lncRNA content was similar between samples, with PCBP1-AS1, TEX41, and PVT1 being the top represented transcripts. There were 286 miRNAs found in the salEV samples. The pathways predicted to be affected by the top represented miRNAs include Hippo signaling, TGF-β signaling, Wnt signaling, FoxO signaling, ErbB signaling, axon guidance, and mTOR signaling. We could detect retrotransposon transcripts from LINE, SINE, and LTR elements in salEVs. When compared to blood-derived EVs, salEVs showed greater representation of transcripts associated with neurodegenerative pathways.

**Discussion:**

Our results indicate that salEVs contain transcripts that are associated with pathways involved in neurodegeneration. The presence of these transcripts in salEVs suggest that saliva may be used to screen for biomarkers of neurodegenerative diseases.

## Introduction

Human saliva contains numerous factors such as proteins, DNA, and RNA. Saliva has been shown to contain many of the same metabolites that are found in blood, likely due to the proximity of the salivary glands to blood vessels allowing for exchange of components between the blood and saliva (Yan et al., 2009). Salivary glands are innervated by the autonomic nervous system, which is influenced by CNS activity. CNS conditions that impact autonomic function can lead to changes in saliva composition, which may reflect neurological alterations in the CNS (Naumova et al., 2014; Nazir, 2024; Proctor and Carpenter, 2007). In addition, there is high correlation between metabolites found in cerebrospinal fluid and saliva (Reuster et al., 2002), making analysis of saliva an attractive, non-invasive screening mechanism for changes in the central nervous system.

The composition of saliva can be affected by the health status of the individual, and the ease of collection has led to saliva being investigated for potential biomarkers of disease (Gardner et al., 2020; Meleti et al., 2020). It was first shown more than a decade ago that circulating tumor DNA (ctDNA) could be detected in the saliva of lung cancer patients (Wei et al., 2014). Since that time saliva has been investigated for biomarkers of several forms of cancer, primarily using omics-based studies. Whole saliva was shown to be a promising substrate for biomarker identification in for oral squamous cell carcinoma detection at an early stage when treatments may be more effective (Esperouz et al., 2024). The miRNA content of whole saliva was shown to distinguish colorectal cancer patients from healthy controls (Krokosz et al., 2025). Breast cancer biomarker detection sensors have been developed for saliva and show promise for detecting these cancers early (Grasso et al., 2025). These findings show that saliva is a valuable source of biomarkers that can allow for disease detection at an early stage.

Several of the cancer biomarkers identified in whole saliva, including proteins and miRNAs, are present in extracellular vesicles (EVs). EVs are small membrane bound particles that are released by most cell types, including cell types found in the central nervous system (Faure et al., 2006; Kramer-Albers et al., 2007; Potolicchio et al., 2005; Taylor et al., 2007). Originally thought to be cellular junk, as a way of the cell taking out the trash, they are now recognized as potent mediators of intercellular communication. Once released from the cell of origin EVs can target cells in the immediate vicinity or can enter the bloodstream and affect cells that are far from the cell of origin. EV cargo includes mRNA, miRNA, other ncRNAs, DNA, lipids and protein in a cell type specific manner that is believed to closely mirror the cytoplasmic content of the cell of origin (Quesenberry et al., 2015; Yokoi et al., 2019). Once an EV reaches its target cell, it can release its cargo into the cytoplasm of that cell (Montecalvo et al., 2012). RNA released into a target cell is translated using the target cell translation machinery, since EVs do not contain ribosomal RNAs (Palanisamy et al., 2010; Valadi et al., 2007). EVs can cross epithelial barriers by transcytosis allowing them to cross from the blood stream into the saliva (Guo and Jiang, 2020; Serra and Sundaram, 2021). This mechanism also allows EVs to cross the blood brain barrier, allowing for accumulation of EVs derived from the central nervous system in blood, which can ultimately lead to their accumulation in saliva.

It was recently shown that the RNA content of salivary EVs (salEVs) can distinguish people who have a traumatic brain injury from those who have not (Cheng et al., 2019; Cheng et al., 2020), highlighting their potential diagnostic value. It has also been shown that Alzheimer’s disease (AD) associated proteins can be detected in saliva (Bermejo-Pareja et al., 2010; Conrad et al., 2002; Lee et al., 2017; Reuster et al., 2002; Sabbagh et al., 2018). In this study we determined that RNA transcripts associated with neurodegenerative pathways can be detected in salEVs in people over the age of 65. We also identified non-protein coding sequences that are associated with neurodegenerative conditions. Detecting changes in salEV RNA content may allow for the discovery of biomarkers of neurodegeneration early in the disease process, allowing for treatments to begin at an earlier point when therapeutics may be more effective. This preliminary study sets the stage for detection of biomarkers of neurodegeneration in salEVs.

## Materials and methods

### Participants

Whole saliva specimens were collected from healthy older (65+) adult participants (N = 15). enrolled in a longitudinal study of neurodegeneration and cognitive aging at the Rhode Island Hospital Alzheimer’s Disease and Memory Disorders Center in Providence, RI USA (Table 1). Healthy volunteers were excluded from the study if they had signs of dementia, major primary psychiatric illnesses, congenital brain disorders, intellectual disability, and history of traumatic brain injury. Informed consent was obtained from participants before enrollment in the study. The Brown University Health institutional review board approved the study in accordance with good clinical practice guidelines and the Declaration of Helsinki.

**TABLE 1:** Study participant characteristics.

Characteristics	Value
Total number of samples	15
Age, (mean), years, (SD)	72 (3.4)
Sex, % female	80
Race and ethnicity, (n)	15 White, non-Hispanic
Montreal cognitive assessment, (mean), total score, (SD)	27 (1.7)
Mini-mental state exam, (mean), total score, (SD)	27.9 (1.9)
APOE proteotype	N
E2/E3	3
E3/E4	4
E3/E3	8

### Saliva collection

Participants were instructed to abstain from eating, chewing gum or smoking at least 2 h before the saliva collection. After signing consent, a research assistant described the procedure and provided a sterile polypropylene collection and storage kit (SputEm™, Simport). Participants were encouraged to produce a minimum of 5 mL saliva. Within 2 min of completion, the collection tube was sealed and stored at −80 °C until analyzed.

### EV isolation

The protocol for salEV isolation was adapted and modified from the extracellular vesicle isolation protocols of Lasser et al. (2011), Michael et al. (2010). Briefly, samples were kept frozen at −80 °C until processed. Frozen saliva was thawed in 37 °C water bath with DNase I (Roche) at 100 µg per milliliter of saliva for 20 min. Collected saliva (5 mL) was diluted with 1x phosphate buffered saline (PBS, Gibco) to a final volume of 25 mL. The saliva suspension was centrifuged at 2000 *g* for 10 min at 4 °C to remove large debris. The supernatant was transferred to another tube and centrifuged at 17,000 x g for 15 min at 4 °C to further remove unwanted organelles and cell fragments. Following the initial centrifugation steps, the supernatant was transferred to sterile 36 mL PA tubes (Sorvall) for ultracentrifugation at 120,000 x g for 70 min at 4 °C in a Surespin630 rotor (Sorvall) with full brake and acceleration (both set at 9). Following ultracentrifugation, the aqueous layer, which is viscous in whole saliva samples, was removed and the pellet containing the EVs was washed with phosphate buffered saline (PBS, Thermo Fisher Scientific) and ultracentrifuged again at 120,000 x g for 70 min at 4 °C. For long term storage at −80 °C, EV pellets were resuspended in 1% dimethyl sulfoxide (DMSO, Sigma) in PBS.

### Measurement of particle size and concentration distribution with NanoSight

Nanoparticles in the salEV suspensions were analyzed using the NanoSight NS500 instrument (NanoSight Ltd). The analysis settings were optimized and kept constant between samples, and each video was analyzed to give the mean, mode, median and estimated concentration for each particle size. Samples were measured at 1:20 dilution, yielding particle concentrations in the range of 1 × 10^8^ particles ml^−1^ in accordance with the manufacturer’s recommendations. All samples were analyzed in triplicate.

### RNA isolation

EV pellets after ultracentrifugation were lysed using Trizol (Invitrogen). RNA was isolated using Trizol according to the manufacturer’s protocol and dissolved in Nuclease-Free water (Ambion). Briefly, samples were centrifuged following the addition of chloroform and the aqueous phase containing RNA was collected. RNA was precipitated using isopropanol and collected by centrifugation at 12,000 x g for 15 min. The RNA pellet was washed with 70% ethanol and resuspended in RNase free water. Quantification was done using a Nanodrop 1000.

### Quantitative RT-PCR

cDNA was synthesized from RNA with the High Capacity cDNA transcription kit (Applied Biosystems) in a final volume of 20 μL. Amplification reactions consisted of one cycle for 10 min at 25 °C, one cycle for 120 min at 37 °C, and one cycle for 5 min at 85 °C using a 9800 Fast Thermal Cycler (Applied Biosystems). Pre-amplification reactions were performed in a final volume of 50 µL consisting of 12.5 µL of diluted 96 TaqMan gene assay mix, 25 µL of TaqMan Preamp Master mix (Applied Biosystems) and 12.5 µL of cDNA. The reaction consisted of a 10 min denaturation step at 95 °C followed by 14 cycles of 15 s at 95 °C then 60 °C for 4 min. TaqMan® Human Alzheimer’s Disease array (cat # 4378713) cards were loaded with cDNA and TaqMan® Universal PCR master mix (Applied Biosystems) and run on the Viia7 Real-Time PCR System (Life Technologies) using Relative Quantification settings. Each sample was run in duplicate. The results were analyzed using the QuantStudio software (Applied Biosystems). A gene was determined to be present if the cycle threshold value (Ct) fell within the dynamic range of the assay (Ct value between 18 and 35) in both runs.

### Protein isolation

SalEVs were lysed on ice for 30 min using 10X cell lysis buffer (Cell Signaling) supplemented with Halt Protease and Phosphatase Inhibitor Cocktail (100X) (Thermo Fisher Scientific) following the manufacturer’s recommendations. The lysates were then centrifuged at 14,000 × g for 15 min at 4 °C, and the supernatant was collected. Protein concentration of EV lysates was quantified using the Pierce™ BCA Protein Assay Kit (Thermo Fisher Scientific).

### Western blots

SalEV lysates were loaded onto 4%–20% Mini-PROTEAN® TGX™ Precast Protein Gels (Bio-Rad) and separated by SDS-PAGE. The proteins were then transferred to PVDF membranes (Bio-Rad) using the Trans-Blot Turbo Transfer System (Bio-Rad). The membranes were incubated overnight at 4 °C with the following primary antibodies: CD81 (System Biosciences, #EXOAB-CD81A-1), CD9 (System Biosciences, #EXOAB-CD9A-1), CD63 (System Biosciences, #EXOAB-CD63A-1), TSG101 (Thermo Fisher Scientific, #MA1-23296), PDCD6IP (Thermo Fisher Scientific, #50-167-7058), heat shock protein 70 (HSP70; Santa Cruz, #sc-59560), and APOA1 (Santa Cruz, #sc-376818). Signal on PVDF membranes were developed with SuperSignal™ West Atto Ultimate Sensitivity Substrate (Thermo Fisher Scientific) and captured using the ChemiDoc™ MP Imaging System (Bio-Rad).

### TEM

Transmission electron microscopy was performed on isolated salEVs fixed with 2% paraformaldehyde, placed on 200 mesh copper formvar carbon coated grids (Electron Microscopy Science) and left to adhere for 20 min. EVs were negatively stained with UAR-EMS (Electron Microscope Science). Grids were viewed on a Philips 410 Transmission Electron Microscope equipped with an Advantage HR CCD camera or on a Thermo Apreo VS SEM.

### RNA sequencing

A total of 5 µL of RNA (regardless of RNA concentration) isolated from salEVs was sent to Azenta for low-input, paired-end RNA sequencing. The fastq files were groomed using fastp (Chen, 2023) to remove adaptors, filter poor quality reads, and remove duplicates using the default settings and enabling base correction (-c), polyX tail trimming (-x), unique molecular identifier processing (-U), and deduplication (-D). The resulting files containing high quality reads were mapped to the human genome HG38 using STAR (Dobin et al., 2013) using default setting except for the multi-mapping set to 100 (--outFilterMultimapNmax 100), the output set to keep only reads that contain junctions that pass filtering into SJ.out.tab (--outFilterType BySJout), and the minimum number of matched bases normalized to read length set to 0.33 (--outFilterMatchNminOverLread 0.33). The identity of the features represented by the mapped reads were determined by FeatureCounts (Liao et al., 2014) utilizing the default settings and the Gencode Release 43 (GRCh38.p13) transcript annotation file. The pathways associated with the identified coding transcripts were determined using the Kyoto Encyclopedia of Genes and Genomes Database for Annotation, Visualization, and Integrated Discovery database (Huang da et al., 2009; Sherman et al., 2022). The RNA was classified by type (i.e., protein coding, lncRNA, etc., …) and transcription factor binding sites were identified using ShinyGO 0.77 utilizing the default settings (Ge et al., 2020). The raw fastq files can be found on NCBI under BioProject PRJNA1380499.

### Comparison of RNAs in blood EV (bEV) and salEV

We used 117 publicly available blood EV (bEV) RNA-seq datasets from healthy control donors that were part of GSE133684 (Yu et al., 2020). The healthy control participants ranged from 41-91 years in the bEV datasets. RNA features that are highly present in each source (salEV or bEV) are separately identified. Specifically, the average of log transformed feature counts in the salEV samples are computed as:

$$\mu_{g}=\frac{1}{n}\sum_{i=1}^{n}\log\left(x_{gi}+1\right)$$

where $x_{gi}$ is the count for feature g in sample $i\;\left(i=1,\ldots ,15\right)$ . The RNA features that rank among the top 1000 in $\mu_{g}$ are identified as the highly represented in salEVs. Similarly, the most abundant RNAs in blood EVs are identified using the average of the log transformed counts among the blood EVs. The composition of the two lists of top 1000 genes is compared, rather than the level of specific RNA features across the two sources. For a pathway of interest that includes k genes, we compare the proportion of these k genes that are among the top 1000 in each source.

### miRNA analysis

The miRNA content of salEVs was determined using the Nanostring platform. Between 12.87 – 100 ng/sample were run on a NanoString Human v3 miRNA Assay at Boston Children’s Hospital IDDRC Molecular Genetics Core. Representation levels of 827 miRNAs were determined using the nCounter software (Nanostring). The mirPath program on the Diana Tools website was used to predict the physiological pathways that would be affected by alterations in the miRNA cytoplasmic concentrations (Vlachos et al., 2015).

### Repetitive element analysis

Expression levels of repetitive elements in salEVs were determined using the TE-seq methods outlined in Kelsey et al. (Kelsey et al., 2025). Briefly, the sequencing files described above for RNA sequencing were groomed with fastp as described above, then aligned to the telomere-to-telomere (T2T) human genome (Nurk et al., 2022; Rhie et al., 2023) using STAR with the default settings except the maximum number of loci anchors allowed to map set to 1000 (--winAnchorMultimapNmax 1000), multi-mapping set to 1000 (--outFilterMultimapNmax 1000), the maximum size of the SAM record set to 800,000 (--limitOutSAMoneReadBytes 800000), the output set to keep only reads that contain junctions that pass filtering into SJ.out.tab (--outFilterType BySJout), and the minimum number of matched bases normalized to read length set to 0.33 (--outFilterMatchNminOverLread 0.33). The alignment files were processed with Telescope to determine the repetitive element counts in each sequencing dataset using the default settings with the GTF attribute set to gene ID (--attribute gene_id) and the reassignment mode set to average (--reassign_mode average) (Bendall et al., 2019).

## Results

### Characterization of EVs isolated from saliva

**FIGURE 1:**
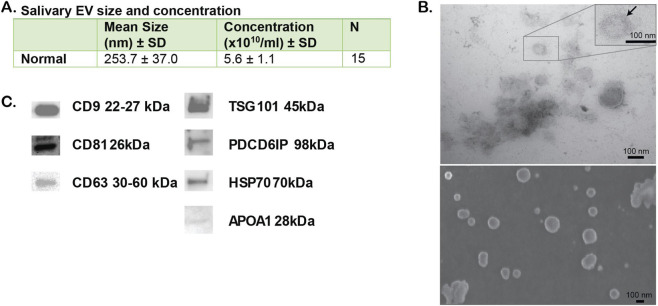
Characterization of salEVs. **(A)** Mean size and concentration of salEVs determined by a Nanosight analysis. **(B)** Transmission electron micrographs and a scanning electron micrograph of a representative salEV sample. Note the presence of a lipid bilayer encompassing the EVs (insert). **(C)** Western blot analysis of salEVs. CD9, CD81 and CD63 are tetraspanins are known EV markers, TSG101 and PDCD6IP are cytoplasmic proteins with membrane binding ability, HSP70 is a common EV cargo protein, APOA1 is associated with lipoprotein particles that commonly contaminate EV preparations.

We collected whole saliva from 15 cognitively normal older adults (mean (SD), 72 (3.4) years) and isolated salEVs. Participants were non-Hispanic White and the majority (80%, n = 12) were female (Table 1). To verify that the isolated particles are EVs, we characterized the vesicles in compliance with the minimal information for studies of extracellular vesicles 2023 (MISEV 2023) (Welsh et al., 2024). We performed Nanosight analysis on the salEV preparation (Figure 1A). The particles in the salEV preparations were present at an average concentration of 5.6 ± 1.1 × 10^10^/mL. Analysis of the size distribution of the salEV particles showed a mean diameter of 253.7 ± 37.0 nm.

To further evaluate that the particles in the preparation are EVs, we used transmission electron microscopy (EM) to visualize the particles. The electron micrographs revealed cup-shaped membrane bound vesicles (Figure 1B). Closer examination of the vesicles revealed that they were enclosed by a double membrane (Figure 1B insert).

To determine that the membrane-bound vesicles seen by EM are EVs, we confirmed the presence of EV-specific markers. We performed Western blot analysis for the transmembrane tetraspanin markers CD9, CD63, and CD81, and found that all 3 tetraspanins were present in the preparations (Figure 1C; Supplementary Figure S1). We also verified the presence of cytosolic proteins with lipid or membrane protein binding ability by confirming the presence of tumor susceptibility gene 101 (TSG101) and programmed cell death 6-interacting protein (PDCD6IP) (Figure 1C; Supplementary Figure S1), both of which play a role in the endosomal sorting complexes required for transport (ESCRT) pathway that produces extracellular vesicles. Heat shock protein 70 (HSP70) is commonly found in EV cargo (Lancaster and Febbraio, 2005), and we verified the presence of HSP70 in the vesicle preparation (Figure 1C; Supplementary Figure S1). As a negative control, we examined levels of APOA1, which was minimally present indicating that the EV preparation was not significantly contaminated with lipoprotein particles (Figure 1C; Supplementary Figure S1). From these data we conclude that our preparations from saliva contained extracellular vesicles.

### Transcriptomic data

We isolated total RNA from the salEV preparations. The overall quality of the RNA was acceptable, considering that EV RNA quality is often considered poor due to the presence of fragmented and degraded RNAs (van Balkom et al., 2015). The RNA had an average 260/280 of 1.7 ± 0.2 and an average total RNA concentration of 20.2 ± 9.0 ng/μL and a small RNA concentration of 5.5 ± 7.7 ng/μL.

#### mRNA characterization

**FIGURE 2:**
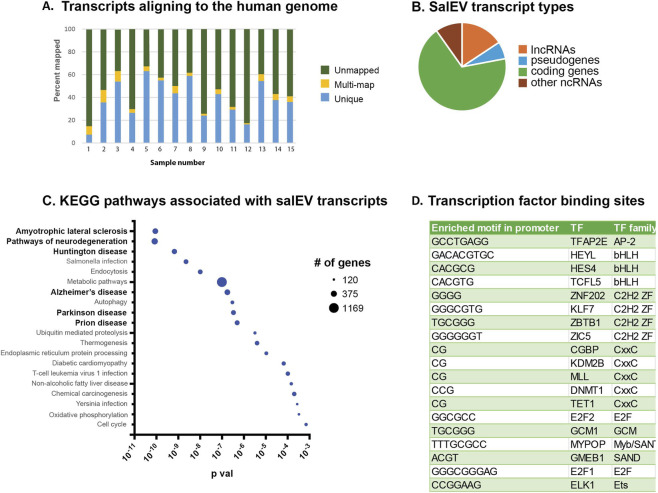
RNA-seq analysis of salEVs. **(A)** The percentage of reads that uniquely or multi-map to the human genome. **(B)** Transcript types found in salEVs. **(C)** KEGG pathways associated with messenger RNA transcripts found in salEVs. Pathways in bold are known to be dysregulated in AD. **(D)** Transcription factor binding sites enriched in promoter regions of mRNA transcripts in salEVs.

To characterize the larger transcripts present (>200 nt) we performed bulk-RNA-sequencing on salEVs from 15 samples. Since saliva is not sterile, and prokaryotic species are known to produce EVs (Mobarak et al., 2024), we examined the number of transcripts mapping to the human genome and the number mapping to bacterial genomes to determine the approximate representation of EVs originating from human cells in saliva. We found an average of 44.1% of the reads uniquely mapped and 4.3% of the reads multi-mapped to the human genome (Figure 2A). The majority of the remaining reads multi-mapped to bacterial genomes (Supplementary Figure S2). These results indicate that a significant portion of salEVs originate from human cells, and there is high enough representation to identify potential biomarkers for disease from salEVs.

Further analysis of the RNA content revealed that for most samples the majority of the transcripts >200 nucleotides originated from protein coding sequences. We examined the transcripts aligning to Ensembl annotations (vertebrate, release 96, (Aken et al., 2017)) and found an average of 19,541 distinct transcripts represented in each salEV preparation (Figure 2B). On average, there were 12,213 protein coding gene transcripts represented in the data, or 68.1%. The next largest category of RNA was long, non-coding RNAs (lncRNAs), which represented approximately 3,457 transcripts in the preparations, or 15.7% of the transcripts. This is interesting since lncRNAs are known to play regulatory roles and the transfer of these transcripts to other cell types could affect cellular processes at distant locations. The remaining transcripts arose from pseudogenes and other non-coding RNAs. We were not able to quantify small RNAs such as miRNAs in these datasets, since the sequencing library preparation included size selection for transcripts >200 nt. Overall, we found a diverse array of RNA species present in salEVs, indicating that salEVs may affect a broad range of physiological processes.

We determined the top KEGG pathways represented by the protein coding transcripts. The top represented pathways were associated with neurodegeneration (Figure 2C). These included Amyotrophic lateral sclerosis, Pathways of neurodegeneration–multiple diseases, Huntington disease, Alzheimer disease, Parkinson disease, and Prion disease. Our results suggest that transcripts associated with neurogenerative conditions are present in salEVs, and that salEVs could be used to screen individuals for risk of developing one of these conditions.

We analyzed the promotor regions of the genes identified in our sequencing results to determine if specific transcription factor (TF) target genes are overrepresented in our data. We found the most significantly represented TF enriched motifs in the promoters of coding transcripts were AP-2ε, which is a member of the AP-2 TF family, and HEYL, HES4, and TCFL5, which are members of the bHLH TF family (Figure 2D). The bHLH TFs have been shown to act as transcriptional repressors and are abundantly expressed during development (Heisig et al., 2012). These results suggest that the salEV cells of origin have increased levels of AP-2ε, and reduced levels of the bHLH TFs.

#### Other RNA types

**FIGURE 3:**
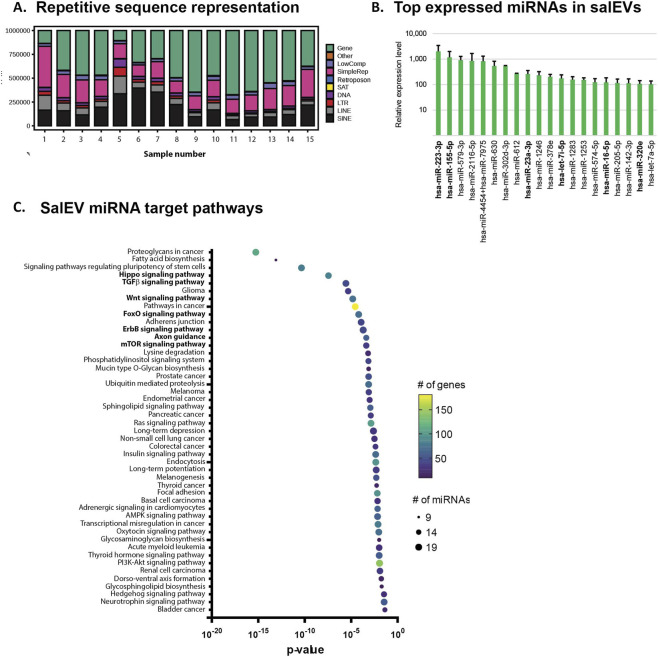
Non-coding RNAs found in salEVs. **(A)** The distribution and types of repetitive elements found in salEVs. **(B)** The top 20 most abundant miRNAs found in salEVs. miRNAs in bold are differentially expressed in AD and inflammation. **(C)** Pathways predicted to be targeted by miRNAs found in salEVs. Pathways in bold are known to be dysregulated in AD.

Repetitive elements represent a large portion of the human genome. While many repetitive elements are structural (centromeric and telomeric repeats), others have evolved from viruses and are capable of transposition within the genome. A subset of the latter type, retrotransposons, have evolved from latent retroviruses that inserted into primordial genomes, and several families contain members that are capable of transposition. We investigated whether retrotransposon sequences are found in salEV cargo. Using the T2T human genome annotation that includes all genomic sequences we found that all 3 classes of retrotransposons were present, including the long interspersed nuclear elements (LINEs), short interspersed nuclear elements (SINEs), and long terminal repeat retrotransposons (LTRs) (Figure 3A). The total number of retrotransposon transcripts present was highly variable between individual samples. Transcript counts for these elements were relatively high, indicating that they may be targeted to the salEVs. Contrary to what is seen in aged tissue and senescent human cell lines, the centromeric satellite sequences did not have significant representation in the salEV RNA content (De Cecco et al., 2013a; De Cecco et al., 2013b). This result also indicates that retrotransposons may be targeted to the EVs, since the centromeric satellite sequences show the greatest increase in expression in aged tissues and senescent cell lines.

We examined the top 20 represented lncRNA transcripts in each sample and found that 16 lncRNAs were among the top 20 in ≥50% of the samples, indicating a high degree of similarity in lncRNA cargo between individuals (Supplementary Figure S3). PCBP1-AS1 was the most abundant lncRNA transcript in all but 1 of the samples. This was followed by TEX41, the second most abundant transcript in 13 samples. PVT1 was present in all 16 samples, and was the most abundant transcript in 1 sample, and the third most abundant transcript in 13 samples. LINC00635, MIR99AHG, MUC20-OT1, CCDC26, SNHG14, HULC, MIR4435-2HG, CASC15, SOX2-OT, LINC01206. SNHG5, MIR100HG, and SNH17 comprised the remaining lncRNAs that are among the most abundant across salEV samples. The similarity of lncRNA representation between samples suggests that lncRNA loading into salEVs may not be stochastic and that specific lncRNAs may be targeted to salEVs.

MicroRNAs (miRNAs) are known to be abundant in EVs. To determine which miRNAs are represented in salEVs, we performed Nanostring analysis on the total RNA isolated from 6 of the samples. We found 286 miRNAs that were expressed above background levels. The top 20 miRNA had average counts of 446.7 per sample and all 20 had counts above the low expression cutoff of 100 counts per sample (Figure 3B). The 2 most abundant miRNAs present were miR-223-3p and miR-155-5p, which are involved in the inflammatory response and are differentially expressed in AD, (Liu and Siadat, 2025; Mancuso et al., 2019). In addition, miR-23a-3p, let-7i, miR-16-5p, and miR-320e were included in the top 20 represented miRNAs. These miRNAs are differentially expressed in AD (Kim et al., 2020; Meng et al., 2014; Sorensen et al., 2016; Weinberg et al., 2015). We used the mirPath software on the Diana Tools website to predict which pathways would be affected by altered expression of the top 20 miRNAs (Vlachos et al., 2015). The most significantly affected pathways contained between 9 and 181 genes that are predicted to be regulated by between 9 and 19 of the top 20 miRNAs (Figure 3C). Several of these pathways are important in cellular function and altered levels of these miRNAs in salEV target cells could affect these cellular processes. These pathways include Hippo signaling, TGF-β signaling, Wnt signaling, FoxO signaling ErbB signaling, axon guidance, and mTOR signaling.

### Comparison to blood EVs

**FIGURE 4:**
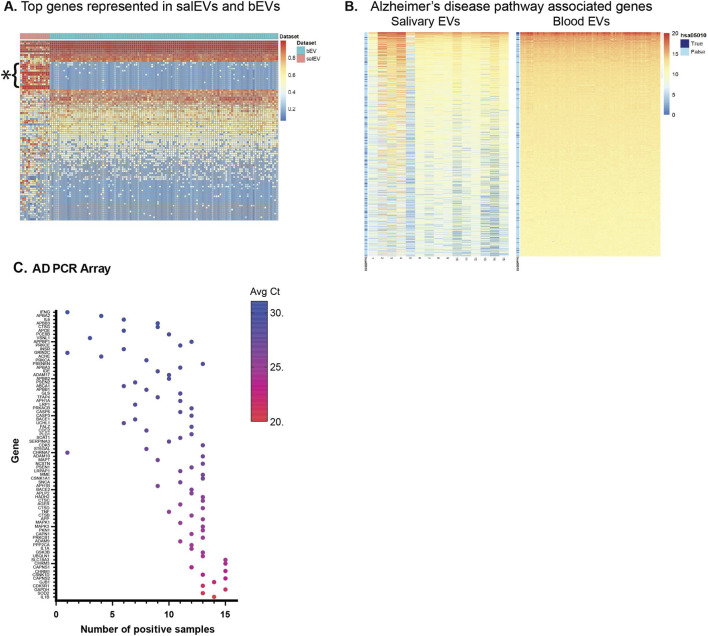
Comparison of salEVs to blood-derived EVs. **(A)** Comparison of the top represented transcripts in salEVs and bEVs. * denotes highly represented genes in salEVs that show lower abundance in bEVs. **(B)** Representation of the top 1000 transcripts found in SalEVs and bEVs. The blue vertical bar to the left of each plot indicates transcripts associated with the Alzheimer’s disease KEGG pathway hsa05010. Pathway transcripts are indicated with a dark blue line. Note the larger number of pathway-associated transcripts in salEVs. **(C)** Distribution of AD specific genes in salEV samples showing most transcripts on the AD array are present in at least 1 salEV sample. Note that lower cycle threshold values (Ct) indicate higher levels of representation in the samples.

We obtained publicly available RNA-seq data from blood derived EVs (bEVs) from 117 healthy donors (Yu et al., 2020). When we contrast the mRNA abundance profiles between salEVs and bEVs, some genes are highly detected in both salEVs and bEVs, but there are a set of genes that are highly present in most salEV samples that are hardly detected in any of the bEV samples (Figure 4A). As expected, many of these genes are related to salivary secretion. What is encouraging is that many genes associated with neurodegenerative diseases are also detected in salEV, and to a greater extent than their presence in the bEVs. Due to the obvious biological difference in the two data source populations (age range, for example,) as well as technical differences (such as isolation method and experimental conditions), a direct comparison between RNA presence level or between salEV and bEV is not meaningful. Instead, we identified the most abundant 1000 RNAs in each source separately and compared the composition of the top 1000 RNAs between the two sources. The top 1000 represented transcripts in salEVs include many that are associated with the Alzheimer’s disease KEGG pathway (hsa05010) (Figure 4B). Specifically, among the 390 genes associated with the AD pathway, 128 (32.8%) are among the top 1000 transcripts found in salEVs. In contrast, only 62 (15.9%) of these genes are among the top 1000 transcripts in bEVs. An extended comparison of six neurodegenerative disease-associated KEGG pathways is shown in Table 2, with the corresponding gene abundance heatmaps in Supplementary Figure S4. For each neurodegenerative condition, there is higher representation of pathway-associated transcripts in salEVs compared to bEVs. Since bEVs have been proposed to be a source of biomarkers for many conditions including cancer and neurodegenerative conditions (Bazan et al., 2025; Torrini et al., 2025), we find the greater presence of neurodegeneration-related genes in salEVs an encouraging sign that makes salEVs a potential source for identifying biomarkers for neurodegenerative diseases, and serve as an effective, non-invasive alternative to bEVs.

**TABLE 2:** Number of genes associated with neurodegenerative pathways.

KEGG	Pathway	salEV (%)	bEV (%)	Pathway size - # of genes
hsa05010	Alzheimer disease	128 (32.8)	62 (15.9)	390
hsa05012	Parkinson disease	124 (45.9)	60 (22.2)	270
hsa05014	Amyotrophic lateral sclerosis	136 (37.2)	71 (19.4)	366
hsa05016	Huntington disease	119 (38.5)	53 (17.2)	309
hsa05020	Prion disease	117 (42.2)	56 (20.2)	277
hsa05022	Pathways of neurodegeneration - multiple diseases	152 (31.6)	84 (17.5)	481

### Validation of bioinformatic data

We validated the presence of AD genes in salEVs by qPCR using the Human Alzheimer’s Disease PCR array designed to detect 94 gene transcripts associated with AD. We found that 78 of the 94 genes (83%) were detected in at least 1 of the 15 samples (Figure 4C). Remarkably, 54 genes (57%) were detected in 10 or more samples. These results confirm that salEVs contain transcripts associated with the AD pathway and that levels of these transcripts in salEVs may be useful for identifying neurodegeneration-related biomarkers.

## Discussion

Our group and others have previously shown that the RNA content of salEVs can distinguish between people who have a traumatic brain injury from those who have not (Cheng et al., 2019; Cheng et al., 2020). These results led us to investigate whether we can identify transcripts that belong to pathways associated with neurodegeneration in salEVs. However, the RNA content of salEVs has not been well described, and in order to identify changes associated with pathology, we must first establish the RNA content found in cognitively normal individuals without disease. To accomplish this goal, we isolated and characterized EVs from saliva from cognitively normal older individuals above 65 years of age.

The idea that EVs originating in the brain can travel to the saliva is further supported by the fact that certain viruses, such as the rabies virus, show primary pathology in the central nervous system and the viral particles are efficiently targeted to the saliva for transmission (Liu et al., 2022). The rabies virus particle size (90 × 180 nm) is similar in size to extracellular vesicles. The presence of EVs originating from the CNS in the saliva suggests that EVs may behave similarly to the rabies virus and spread centrifugally from the CNS to the salivary glands. The exact mechanism of how the rabies virus and CNS EVs are targeted to the saliva is poorly understood. There are several theories on how EVs originating in the CNS can travel to the saliva. One possibility is through the lymphatic system. The recently discovered glymphatic system, in which cerebrospinal fluid and interstitial fluid travel through the brain parenchyma and drain either back into the CSF or into the meningeal and cervical lymphatic vessels (Eide et al., 2018), could provide a mechanism of localization to the salivary glands. The cervical lymphatic vessels drain into the cervical lymph nodes, several of which are embedded in the parotid salivary gland or are in close proximity to the submaxillary salivary gland (Ioachim et al., 1988). It is possible that EVs released into the interstitial space in the brain parenchyma can be washed into the cervical lymphatic vessels and end up in the cervical lymph nodes present near the salivary glands and pass into the saliva. It is also possible that the EVs can travel to the salivary gland via the cranial nerves that innervate these glands. EVs have also been shown to cross the blood brain barrier and enter the circulatory system, which could result in their accumulation in the saliva by transcytosis from the blood (Banks et al., 2020). The exact mechanism that results in accumulation of CNS EVs in the saliva is an area for future investigations.

It was previously reported that a large proportion of extracellular RNA (exRNA) found in saliva is of microbial origin (Kaczor-Urbanowicz et al., 2018). However, this report included all exRNA, not just what is present in salEVs. Kaczor-Urbanowicz and colleagues found that approximately 17% of reads mapped to human genome, we found a greater number of reads mapped to the human genome, with ∼48.4% mapping uniquely or multi-mapping to the human genome. The isolation of the salEVs resulted in a reduction in RNA originating from bacteria and other species present in the oral cavity, likely due to the contaminating RNA being either free floating or present in complexes that were removed with the lower speed centrifugation we used prior to the ultracentrifugation to isolate the EVs. By isolating the EVs from saliva we are enriching for RNA of human origin, allowing us to identify specific transcripts that may indicate a pathology or disease.

We analyzed the RNA content of EVs isolated from saliva to determine the types of transcripts present in this vesicle type. We found that a large proportion of the RNA transcripts mapped to coding genes. The 2023 release of NextProt reveals that the human genome contains 20,389 protein coding genes (Zahn-Zabal et al., 2020). We found an average of 12,213 protein coding genes represented in the transcriptomic data from salEVs, indicating that approximately 60% of the coding genes present in the genome were represented in salEVs. This broad presence of protein-coding genes in salEVs suggests EVs could be a valuable source for identifying biomarkers linked to different diseases or conditions.

Despite the large percentage of represented genes, analysis of the regulatory regions of these genes indicates that many of the transcripts may be targeted to salEVs rather than stochastically packaged into the EV. Analysis of transcription factor binding motifs in the promoter regions of transcripts found in salEVs revealed several binding motifs that were highly represented in the data (Figure 2D). AP-2ε was the most abundant transcription factor binding motif found in transcripts isolated from salEVS. AP-2ε has been shown to play a role in development and has been studied in the context of cancer where it may play a role in regulating apoptosis (Hoshi et al., 2017; Yang and Zhao, 2021), however its role in normal adult cellular processes has not been examined. Our results indicate that AP-2ε is active in the cells of origin in older adults and AP-2ε may influence the cellular physiology of target cells through the transfer of AP-2ε regulated genes.

Binding motifs for three members of the bHLH TF family were also identified as abundant in the regulatory regions of represented protein coding genes found in salEVs. These include HEYL, HES4, and TCFL5. These bHLH TFs are expressed during development and have been shown to regulate multiple developmental pathways, including the differentiation of neural progenitor cells into neurons (Jalali et al., 2011). These TFs have been shown to modulate transcription of their target genes by mostly downregulating their target transcripts rather than creating an on/off process (Heisig et al., 2012). Therefore, the fact that we see transcripts containing the bHLH TF binding sites is not surprising, since the salEVs were collected from older adults where developmental pathways would not be expected to be active. These results suggest that these TFs are not being actively expressed in the cells of origin of the salEVs since these TFs downregulate expression of their gene targets, and we see these transcripts enriched in salEVs in older adults.

Our analysis showed that salEVs contain large numbers of lncRNAs, and that the lncRNAs present were similar across different samples from different individuals, suggesting that these lncRNAs may be preferentially packaged into the EVs. The most abundant lncRNAs found in human cells, such as MALAT1 and NEAT1 (Ji et al., 2003), are not highly represented in salEVs, further supporting the idea that the lncRNAs present may be selectively targeted to salEVs. There are currently more than 20,000 lncRNAs annotated in the human genome and efforts are underway to expand on that number (Frankish et al., 2023; Kaur et al., 2024). Despite the large number of annotated lncRNAs, very little is known about the function of the majority of the lncRNAs. The most abundant lncRNAs in most of the salEV samples were PCBP1-AS1, TEX41, and PVT1. PCBP1-AS1, has been studied in the context of cancer, where it either promotes or inhibits cancer progression depending on the cancer type (reviewed in (Wu et al., 2023)). Interestingly, human tissue expression studies show that PCBP1-AS1 has higher expression in the brain than in many other tissues (Fagerberg et al., 2014), however the role it plays in normal brain function and neurodegeneration has not been studied. TEX41 has also been studied in the context of cancer where it may play a role in regulating autophagy by increasing Runx2 levels (Li et al., 2023), and may control cancer cell proliferation and migration by regulating miR153-3p levels (Yang and Chen, 2023). It may also play a role in calcific aortic stenosis (Vassiliou et al., 2024). The role of TEX41 in the healthy brain and in neurodegeneration has not been studied. PVT1, on the other hand, has been shown to be upregulated in the brain in several pathological conditions, including ischemic stroke and glioblastoma (Han et al., 2019; Lu et al., 2020; Zhang et al., 2019). It will be interesting to determine if levels of these lncRNAs correlate with neurodegenerative changes in the brain and if they can be used as biomarkers of these conditions.

miRNAs are abundant in EVs from all cell types, and we examined the miRNAs present in salEVs. We found 6 miRNAs present in salEVs that are differentially expressed in inflammation and AD (Figure 2B), suggesting that levels of these miRNAs in salEVs may be used as biomarkers for these conditions. We examined the pathways predicted to be affected by the top 20 miRNAs found in salEVs (Figure 3C). Perturbation of these pathways has been shown to occur in several neurodegenerative conditions. Hippo signaling is known to regulate neural stem cell differentiation, including neuronal differentiation and glial differentiation (reviewed in (Ouyang et al., 2020)). TGF-β signaling is involved in anti-inflammatory responses and has neuroprotective effects, and TGF-β deficiency promotes Aβ deposition and neuronal loss in a mouse model of AD (Tesseur et al., 2006). Wnt signaling is dysfunctional in AD, leading to neuropathological conditions (Arredondo et al., 2020). FoxO signaling is involved in the downregulation of IGF1 in AD (Kang et al., 2022). mTor is involved in activation of NF-ϰB that triggers the expression of chemokines and inflammatory cytokines (Singh et al., 2024). These results show that miRNAs that affect pathways that may contribute to neurodegeneration can be found in salEVs, and investigating the miRNA content of salEVs may reveal risk of neurodegenerative changes before the onset of cognitive impairment.

We found a significant representation of transcripts that originated from repetitive regions of the genome. Specifically, there were large numbers of transcripts from retrotransposons. These sequences arose from retroviruses that inserted into a primordial genome (Boeke and Stoye, 1997). Most transposable elements in the genome are inactive due to the accumulation of mutations that have occurred over evolutionary time. However, a few transcripts are intact and encode the machinery necessary for insertion into novel locations in the target cell genome, leading to potential mutations, genomic rearrangements, and genomic instability (Huang et al., 2012). In addition, retrotransposon expression has been linked to activation of the innate immune response resulting in inflammation (De Cecco et al., 2019). Interestingly, retrotransposons have been shown to increase in expression with age (De Cecco et al., 2013a), and we see significant representation of these sequences in salEVs isolated from individuals over the age of 65. This age-associated increase is further augmented in neurodegenerative conditions (reviewed in (Frost and Dubnau, 2024)). For example, the long terminal repeat retrotransposons are elevated in TDP43 pathologies (Douville et al., 2011); and tauopathies, including Alzheimer’s disease, are thought to drive neurodegeneration by activating retrotransposons (Guo et al., 2018; Ochoa et al., 2023; Ramirez et al., 2022; Sun et al., 2018). In addition, inhibitors of the long interspersed nuclear elements and short interspersed nuclear element retrotransposons protect against neurodegeneration and cognitive decline in multiple aging model systems (Wahl et al., 2023). We found a significant number of retrotransposon transcripts are present in salEV cargo in individuals over the age of 65, which leads to the question of whether retrotransposon transcripts are spread to target cells through EVs resulting in the spread of age-associated inflammation and other degenerative processes.

Recently, bEVs have been investigated for potential biomarkers of disease, including cancer and neurodegenerative conditions (Bazan et al., 2025; Torrini et al., 2025). In this study we found greater representation of neurodegeneration-related transcripts in salEVs (Figure 4), indicating that salEVs may be a better source for identifying biomarkers of neurodegenerative diseases than bEVs. For 6 different neurodegenerative disease KEGG pathways, salEVs had approximately twice the number of pathway-associated transcripts than what was found in bEVs (Table 2). Due to the greater representation of neurodegeneration-related transcripts, and the presence of other transcripts such as retrotransposons that are known to be associated with these conditions, salEVs may serve as an effective, non-invasive alternative to bEVs.

Neurodegenerative conditions, including AD, are progressive diseases where pathological proteins build up over many years during a clinically silent phase. Once the onset of symptoms begins, the pathology is well-established and is detectable by current clinical tests. However, once the pathology has advanced to the point of symptom development, current treatments can only slow the progression of the disease process, and the neurodegeneration cannot be stopped or reversed. Therefore, identifying biomarkers of neurodegeneration that can be detected during the prodromal phase are needed so that treatments can begin earlier, possibly at a point when the disease process can be stopped or reversed. In this study we showed that transcripts associated with neurodegenerative processes can be detected in EVs isolated from saliva. Future work will investigate whether these transcripts can identify individuals at the earliest stages of neurodegeneration.

Our study highlights the potential utility of salEVs as biomarkers of neurodegeneration; however, we acknowledge several limitations that may influence interpretation of the results. The modest sample size and cross-sectional design do not allow inference of causal relationships between salEV RNA transcripts and neurodegenerative processes. Interindividual factors such as differences in oral microbiota or unrecognized health-related variables may also have contributed to variability in RNA profiles. In addition, this preliminary work focused exclusively on cognitively normal older adults over the age of 65, which does not permit generalizability to younger populations and those diagnosed with mild cognitive impairment or Alzheimer’s dementia. Future work should include individuals with other neurodegenerative conditions, as we were unable to evaluate whether our observations are specific to Alzheimer’s-related neuropathology or represent broader mechanisms of neuronal injury. Finally, although transcriptomic analyses identified pathways and regulatory elements previously associated with neurodegeneration, prediction of future cognitive outcomes was beyond the scope of this research.

## Conclusion

The data presented in the manuscript show that the RNA cargo of salEVs contain transcripts that are associated with neuronal function. By analyzing these transcripts, we may be able to determine if an individual is at a heightened risk for developing a neurodegenerative condition. Saliva can be collected in a non-invasive manner at a point-of-care facility without any specialized training, increasing access to screening for these debilitating conditions. Future studies will focus on identifying transcripts that indicate if someone may be a candidate for more in-depth testing and treatments to slow the progression of these devastating neurodegenerative diseases.

## Data Availability

The data presented in the study are deposited in the NCBI repository, accession number PRJNA1380499.

## In memoriam

This manuscript is dedicated to the memory of our co-author Peter Quesenbery, MD, who passed away on November 13, 2025.
