# Supplementary figures and images for: RNA transcripts in salivary extracellular vesicle cargo isolated from aged populations
Source: Front Aging. 2026 Jan 12;6:1707720. doi: 10.3389/fragi.2025.1707720 (PMC12832735; doi:10.3389/fragi.2025.1707720)

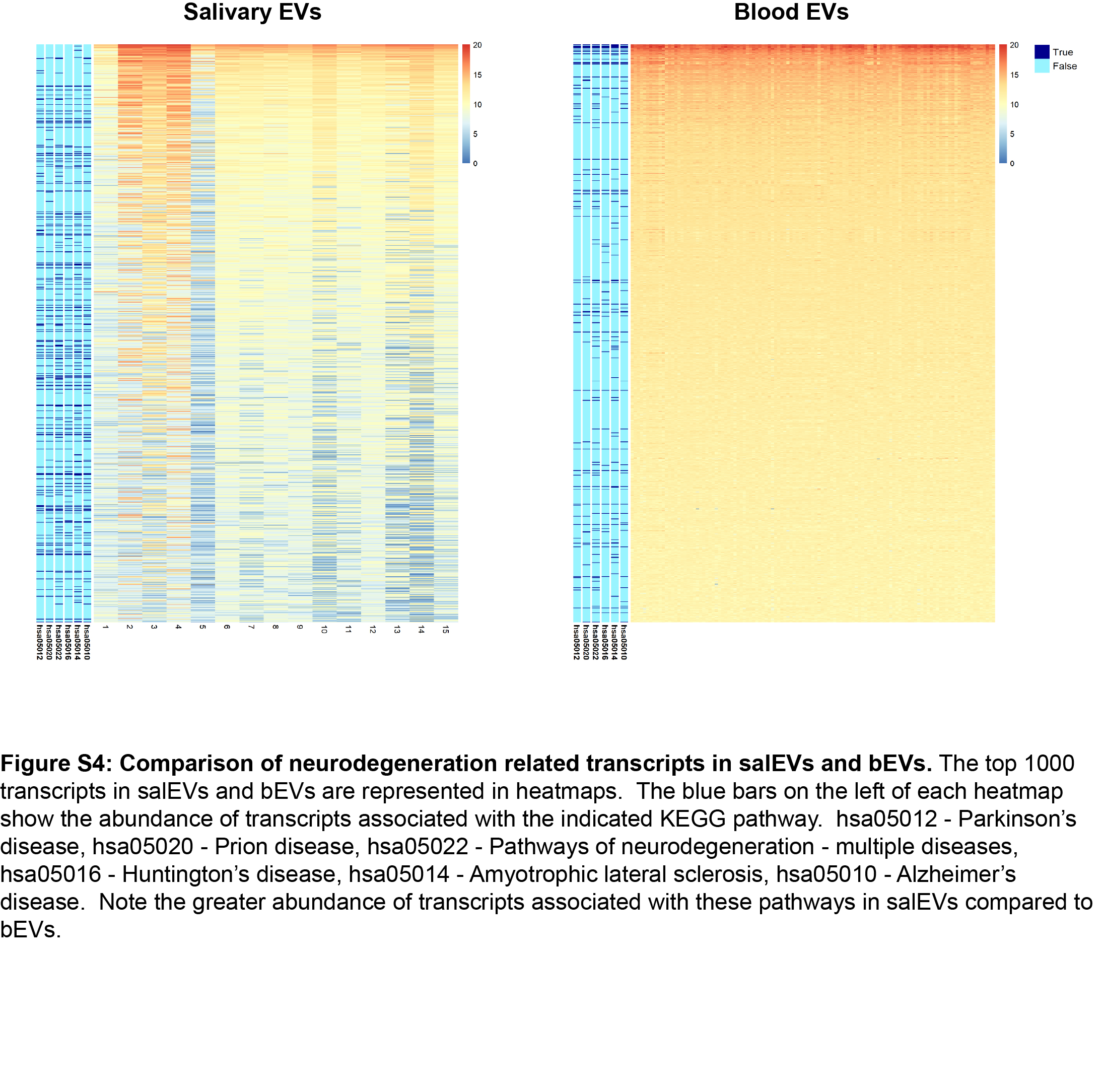

Supplement: Supplementary file 1 [file Image4.jpeg]

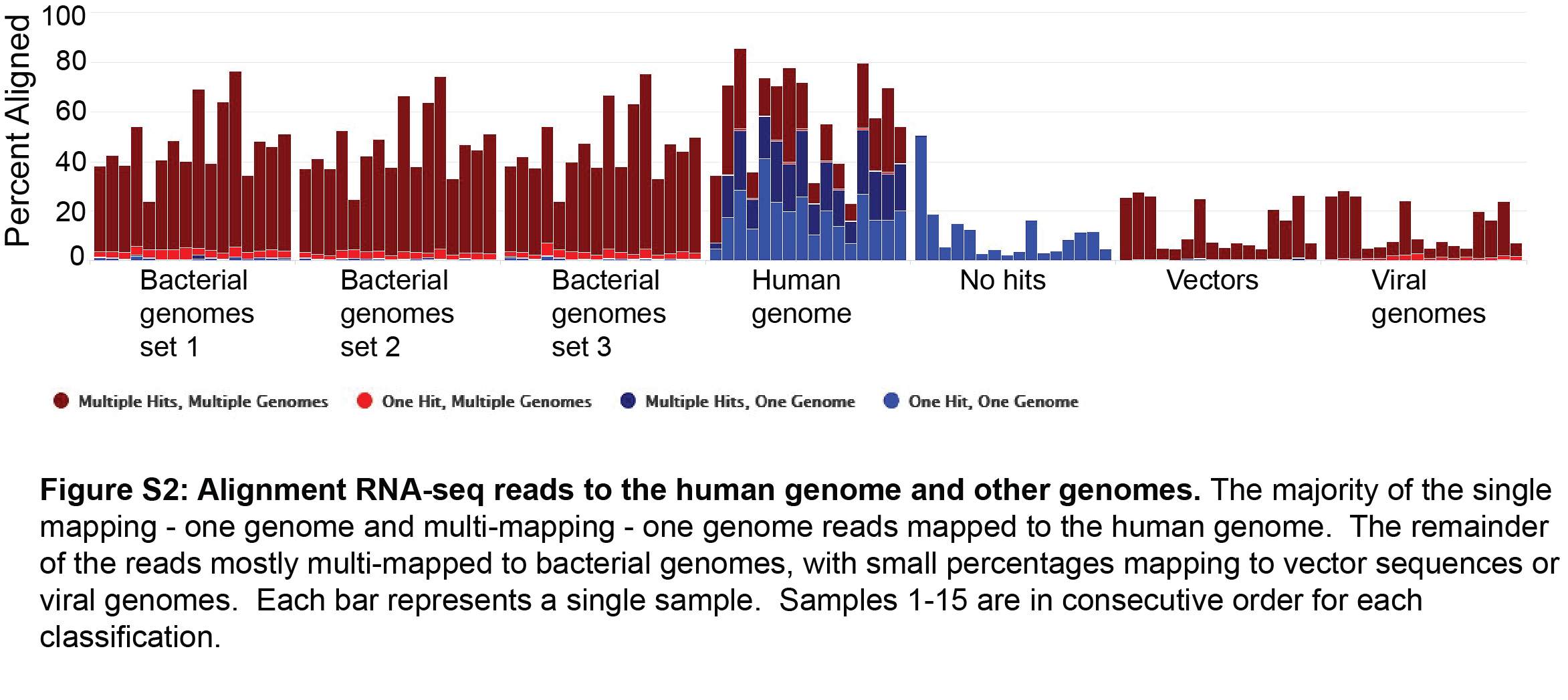

Supplement: Supplementary file 2 [file Image2.jpeg]
